# Continuous Spinal Anaesthesia for Intertrochanteric Femur Fracture in a Patient with Skeletal Dysplasia

**DOI:** 10.1155/2021/6644894

**Published:** 2021-04-09

**Authors:** Sharad Khakurel, Rupesh Kumar Yadav

**Affiliations:** National Academy of Medical Sciences, National Trauma Centre, Kathmandu, Nepal

## Abstract

The practice of continuous spinal anaesthesia is not common. Though underutilised, it offers significant advantage when compared to the single-shot technique nonetheless. Time and again, it has proven its worth in patients with advanced cardiac illness, spinal deformities, and obesity. We here successfully employed this neuraxial anaesthetic technique in a sixty-two-year-old male patient with skeletal dysplasia, who presented for surgical fixation of intertrochanteric fracture of the femur. With short stature, anticipated difficult airway, and poor pulmonary status complicating the anaesthetic plan, we opted for continuous spinal anaesthesia. The procedure was carried out uneventfully with 8 mg of hyperbaric bupivacaine used in titration to anaesthetic needs. Patients with skeletal dysplasia present with wide array of clinical conditions that pose a formidable challenge to anaesthesiologists. Continuous spinal anaesthesia can be safely practiced in such patients as it provides a titratable form of neuraxial blockade with reduced dose of local anaesthesia. This, in turn, ensures a predictable block and, thus, hemodynamic stability.

## 1. Introduction

Skeletal dysplasia encompass a heterogeneous group of disorders that affect the development of bones and cartilage. Its estimated incidence is approximately 15.7 in 100,000 births and most occur as a result of genetic defects. It commonly presents during childhood and is suspected when a patient has short stature, bony deformities, and recurrent fractures [1]. Consequently, the aberrant growth of the long bones, spine, and skull bones leads to morphological abnormalities in the body. Although a disorder of musculoskeletal system, they can also be complicated by involvement of cardiorespiratory and neurologic systems [2].

Patients with skeletal dysplasia may often present with fractures requiring surgical correction. Anaesthetic management of such patients is not without challenges. Deformed skull and facial bones along with vertebral anomalies present with an exacting airway scenario [3–7]. Spinal stenosis and cervical spine instability are common in these patients and warrants extreme caution, especially during laryngoscopy in order to prevent cervical cord compression [3–7]. Thoracic kyphoscoliosis, chest wall deformities with pulmonary involvement, and recurrent respiratory infections are common [7] which makes general anaesthesia a less-preferred option. The spine is also affected by various degrees of kyphoscoliosis and lumbar lordosis; thus, performing neuraxial anaesthesia may become technically difficult [3, 8]. We discuss here an anaesthetic management of one such patient planned for proximal femoral nailing by the use of Continuous Spinal Anaesthesia (CSA).

## 2. Case Presentation

**Figure 1:**
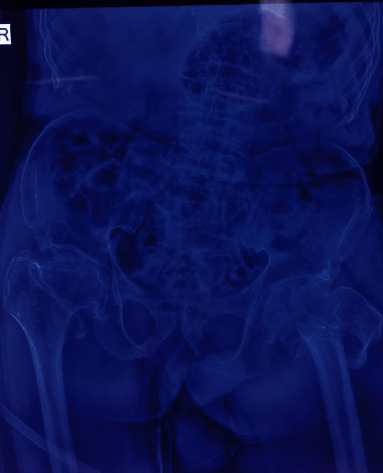
Intertrochanteric fracture of the femur (left).

**Figure 2:**
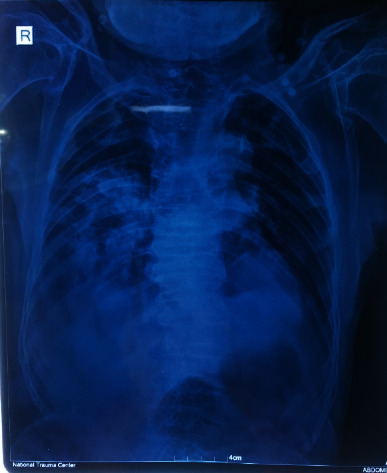
Chest X-ray.

A 62-year-old male was referred to our centre after sustaining injury to his left leg in a landslide. On arrival in the emergency department (ED), his Glasgow Coma Scale (GCS) was 15 with a pulse rate of 88 beats per minute and blood pressure of 120/80 millimetre (mm) of mercury (Hg). The oxygen saturation in room air was 85% with respiratory rate of 20 per minute. He was subjected to trauma imaging, and an X-ray of his left pelvis revealed intertrochanteric fracture of femur (Figure 1). A workup for the patients low oxygen saturation was performed, as there was no history of acute or chronic respiratory illness. On auscultation, there were diminished breath sounds on the right chest. Chest radiography was performed which showed heterogeneous infiltrations in his right lung, with crowding of the ribs and scoliotic spine with narrow intervertebral space (Figure 2). A nasopharyngeal swab for Reverse Transcriptase Polymerase Chain Reaction (RT PCR) for Severe Acute Respiratory Syndrome Coronavirus 2 (SARS-CoV-2) was collected. He was managed in the ED with oxygen via a nasal prong at 3 litres per minute, antibiotics, analgesics, and incentive spirometry and skin traction on the affected limb. The patient was planned for operative intervention and stabilisation of the fracture by proximal femoral nailing.

**Figure 3:**
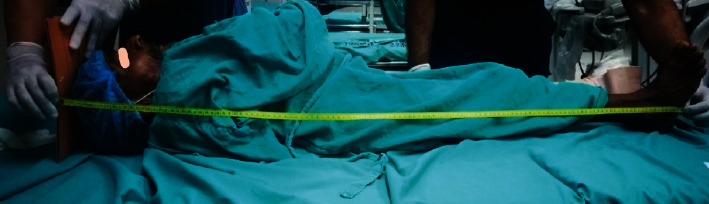
Short stature of the patient.

**Figure 4:**
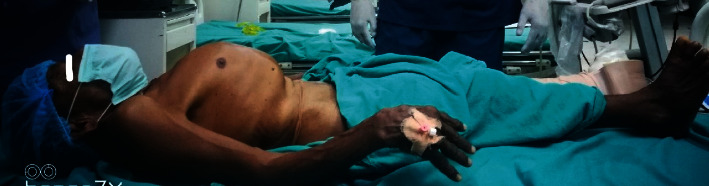
Obvious chest deformity.

During the preoperative assessment of the patient, it was revealed that the patient was a known case of skeletal dysplasia. No further inquiry to ascertain the type of dysplasia was conducted previously. Both his son and daughter also suffered from skeletal dysplasia. The patient was a farmer by profession and had good functional status with a metabolic equivalent of more than four. He had a short stature with a body length of 127 centimetres (cm) (Figure 3) and weight of 45 kilograms (kg). On airway evaluation, the patient had adequate mouth opening with an interincisor distance of 4 cm, Mallampati class III, and missing multiple teeth. He had a short neck with a sternomental distance of 10 cm and thryomental distance of 4.5 cm. On systemic examination, a chest deformity, pectus carinatum, (Figure 4) was visible with a small rib cage. His spine was also examined and had dorsolumbar scoliosis with narrow intervertebral space. Rest systemic examination findings were within normal limits. The lab reports were also within normal limits. A pulmonary function test (PFT) was sought with restrictive lung disease in mind, and an echocardiography was ordered to assess the effect of restrictive lung disease on the heart. Echocardiography showed mild tricuspid regurgitation and mild pulmonary arterial hypertension with a pulmonary artery systolic pressure of 44 mm of Hg with an ejection fraction of 60%. Due to technical issues, the PFT could not be performed.

The patient was planned for regional anaesthesia considering the difficult airway and pulmonary issues. CSA was opted following counselling of the patient and after acquisition of informed written consent. On the day of surgery, the patient was shifted to the operating theatre, and standard American Society of Anaesthesiologists (ASA) monitoring was performed. Injection (Inj.) cefuroxime 750 grams was infused as a part of the infection prevention protocol. A paediatric Tuohy epidural needle 19 gauge was used in an attempt to access subarachnoid space. The procedure, however, was technically very challenging due to positional pain and spine anatomy. During the needling, a whitish discharge was noted from the skin puncture site, which was sent for microbiological examination, and the procedure was abandoned. A spine MRI was performed for further evaluation. It, however, did not reveal any infective pathology, but there was diffuse disc bulge along with disc desiccation of all visible intervertebral discs. A dorsolumbar scoliosis with cervico-dorsal-lumbar spondylosis was also noted.

After observation for 48 hours, the patient was scheduled once again for the contemplated procedure, as microbiological examination was negative for any infective pathology.

In the theatre, the patient was the given Inj. midazolam 1 mg and Inj. fentanyl 50 mcg. Ultrasound guided lumbar plexus block and trans-gluteal sciatic nerve block were performed in the lateral position to provide analgesia for positioning of the patient for CSA and also as a backup in case the planned spinal anaesthesia failed. After twenty minutes of the block, the patient was positioned for spinal anaesthesia in the sitting position. This time, the numeric rating scale for positional pain was 3 out of 10. With the help of a paediatric Tuohy needle, the dura was punctured at 3 cm depth and free flow of the cerebrospinal fluid (CSF) was observed. Then, a paediatric epidural catheter 22 gauge was inserted and fixed at 6 cm. Backflow of CSF was checked, and 0.4 millilitres (ml) (2 mg) of hyperbaric 0.5% Bupivacaine was administered over a minute. The effect of spinal anaesthesia was confirmed on the patient's normal leg. Approximately after eight minutes, the level of sensory block reached T8. Surgery was then allowed to commence as the motor blockade measured by the modified Bromage score reached 2. During the two hours of surgical duration, a single episode of hypotension was noted which resolved with fluid bolus. Based on the assessment of block height regression, the patient received further three aliquots of 0.4 ml of hyperbaric bupivacaine. So, the entire procedure was completed with 8 mg of 0.5% hyperbaric bupivacaine. We removed the catheter in the postoperative unit after infusing another 0.4 ml of hyperbaric bupivacaine. The catheter was removed as the expertise to handle CSA in the postoperative ward was inadequate. The patient was discharged from postoperative care after 24 hours of uneventful stay.

## 3. Discussion

Hip fractures are commonly managed surgically under neuraxial or general anaesthesia (GA). While both techniques have their pros and cons, convincing evidence of one's superiority over the other is lacking. A Cochrane review pointed out the only benefit of neuraxial anaesthesia over GA lies in lowering the risk of deep vein thrombosis, when anticoagulant prophylaxis is not used [9]. The International Consensus on Anaesthesia-Related Outcomes after Surgery review on anaesthesia for elective hip arthroplasty recommended neuraxial anaesthesia in terms of better perioperative outcomes [10].

Single-shot spinal anaesthesia (SSA) and continuous epidural anaesthesia (CEA) are frequently used neuraxial approaches for management of hip fractures. Continuous spinal anaesthesia (CSA), though underutilised, is a well-established neuraxial technique. It safely offers reliable and dense anaesthesia with small doses of local anaesthetic that can be easily titrated. As a result, the block is well predicted and tends to cause less hemodynamic perturbations [11, 12]. Hence, it forms the basis for usage of CSA in high-risk cases [11, 13] and allows CSA to be still in the game and in par with SSA and CEA. CEA on the contrary provides a segmental neural blockade and requires high volumes of local anaesthetics (LA). There always remains a concern for systemic LA absorption and toxicity. Also, when it comes to surgeries involving the hip, which require good muscle relaxation, CSA has shown to provide better block characteristics [11]. With substantial advantages, CSA has also made its way in labour analgesia practice as well [14].

CSA was employed in this case considering the ill effects GA may have on this patient with pre-existing poor pulmonary status. Also, the challenges associated with the difficult airway of this patient could be avoided with a successful neuraxial block. The anaesthetic management of this patient demanded a sound anaesthesia with minimal cardiovascular and respiratory interference. So, undertaking CSA would meet these goals by its ability to titrate the LA with the level of blockade. Various studies have reported better hemodynamic conditions with CSA in comparison to SSA for hip fracture repair [15, 16]. The presence of an indwelling catheter would also allow for LA to be topped up in case of extended duration of surgery and postoperative pain management.

Spinal deformities in the form of accentuated lumbar lordosis, thoracic scoliosis, and narrowed epidural and intrathecal spaces may be encountered in patients with skeletal dysplasia [3, 17]. These deformities pose a hindrance to a successful neuraxial block, thereby increasing the risks of complications such as repeated dural puncture, unpredictable LA spread, high block level, and difficult catheter placement [4–7, 18]. These anatomical features along with short stature make determination of appropriate volume and dose of LA difficult. So, a titratable form of neuraxial anaesthetic techniques such as an epidural, combined spinal epidural (CSE), or continuous spinal may prove to be more suitable in such cases [18, 19].

Once the catheter was in place, we used a low volume of hyperbaric bupivacaine considering the uncertainty lying behind the drug doses and its spread in our patient. Crawford and Dutton used 1 mL of 0.5% plain bupivacaine in a patient with achondroplastic dwarfism titrating it over 30 to 45 min to achieve a T4 level blockade [19]. Dresner and Maclean successfully provided CSA for a caesarean section in a patient with Klippel–Feil syndrome with severe kyphoscoliosis. They used a total of 6.25 mg heavy bupivacaine, 7.5 mg of plain bupivacaine, and 10 mcg of fentanyl over 20 min, without any significant effects on cardiorespiratory function [20]. Okutomi et al. used a total of 7 mg of hyperbaric bupivacaine in titration for a caesarean section using CSA [21]. This evidence favours towards a low volume induction technique in conjunction to intraoperative LA titration, when uncertainty prevails as in this case.

## 4. Conclusions

Continuous spinal anaesthesia is a versatile technique of neuraxial anaesthesia and an indispensable armamentarium of anaesthesiologists. Its inherent potential to provide superior neural blockade even with small doses of a local anaesthetic ensures delivery of safe, reliable, and well-controlled anaesthesia, especially in situation like ours where hemodynamic stability is crucial.

## Data Availability

The data used to support findings of this study are included in the article.

## Abbreviations

CSA: Continuous spinal anaesthesia
ED: Emergency department
GCS: Glasgow coma scale
Mm of Hg: Millimeters of mercury
RT PCR: Reverse transcriptase polymerase chain reaction
SARS-CoV-2: Severe acute respiratory syndrome coronavirus 2
Cm: Centimetres
kg: Kilograms
PFT: Pulmonary function test
ASA: American Society of Anaesthesiologists
Inj: Injection
Mg: Milligram
Mcg: Microgram
CSF: Cerebrospinal fluid
Ml: Milliliters
GA: General anaesthesia
SSA: Single-shot spinal anaesthesia
CEA: Continuous epidural anaesthesia
LA: Local anaesthesia
CSE: Combined spinal epidural.
